# ADIBO-Based “Click” Chemistry for Diagnostic Peptide Micro-Array Fabrication: Physicochemical and Assay Characteristics

**DOI:** 10.3390/molecules18089833

**Published:** 2013-08-16

**Authors:** Denis Prim, Fabien Rebeaud, Vincent Cosandey, Roger Marti, Philippe Passeraub, Marc E. Pfeifer

**Affiliations:** 1 Institute of Life Technologies, University of Applied Sciences Western Switzerland, Sion 1950, Switzerland; E-Mails: denis.prim@hevs.ch (D.P.); fabien.rebeaud@gmail.com (F.R.); vincent.cosandey@hotmail.com (V.C.); marc.pfeifer@hevs.ch (M.E.P.); 2 Institute of Chemistry, University of Applied Sciences Western Switzerland, Fribourg 1705, Switzerland; E-Mail: roger.marti@hefr.ch; 3 Institute of Sciences and Industrial Technologies, University of Applied Sciences Western Switzerland, Geneva 1202, Switzerland; E-Mail: philippe.passeraub@hesge.ch

**Keywords:** peptide microarray, antibody diagnostics, SPAAC, ADIBO, kinetics, non-specific binding (NSB), blocking reagents

## Abstract

Several azide-derivatized and fluorescently-labeled peptides were immobilized on azadibenzocyclooctyne (ADIBO)-activated slide surfaces via a strain-promoted alkyne-azide cycloaddition (SPAAC) reaction revealing excellent immobilization kinetics, good spot homogeneities and reproducible fluorescence signal intensities. A *myc*-peptide micro-array immunoassay showed an antibody limit-of-detection (LOD) superior to a microtiter plate-based ELISA. Bovine serum albumin (BSA) and dextran covalently attached via “click” chemistry more efficiently reduced non-specific binding (NSB) of fluorescently-labeled IgG to the microarray surface in comparison to immobilized hexanoic acid and various types of polyethylene glycol (PEG) derivatives. Confirmation of these findings via further studies with other proteins and serum components could open up new possibilities for human sample and microarray platform-based molecular diagnostic tests.

## 1. Introduction

Immobilization of biomolecules on solid surfaces in a microarray format allows high-throughput screening for interaction partners. Current applications encompass both basic and clinical research as well as molecular diagnostics [1]. Microarray platforms provide multiple advantages over classical microtiter plates, such as minimal reagent and sample consumption, multiplex analysis capability, rapid result generation, suitability for automation and reduced costs. Key aspects in the manufacturing of microarrays are to ensure that immobilized biomolecules maintain their biochemical properties and that immobilization on the surface is robust enough so that they are not removed from the surface by washing steps during the test procedure [2]. The first *in-situ* construction of a peptide microarray was published in a landmark article twenty years ago [3]. Displaying synthetic peptides on a solid surface is an attractive technology to detect ligands, such as antibodies, and has already been developed for various applications, including diagnosis of cancers and infectious diseases, epitope mapping, drug discovery and protein profiling [4,5,6,7,8,9,10]. Today, synthetic peptides are easily produced and purified as well as chemically modified enabling fast and straightforward site-specific and orthogonal immobilization on solid surfaces. A variety of surface chemistries and immobilization strategies are available to covalently attach peptides. Selecting the right strategy is important to overcome limitations of passive adsorption or random binding via amine or sulfhydryl groups that may lead to weak binding and inadequate presentation of the peptide to its substrate. The classical methodology is the attachment via amide/peptide bond formation. Another widely used approach is by reaction of aldehyde-functionalized peptides obtained by periodate oxidation of an N-terminal serine with hydrazine functionalized surfaces [11]. However, these methods are not always compatible with the high-density peptide microarray manufacturing requirements and are often associated with quality issues or significant costs [12,13]. In preliminary work, we evaluated the functionalization of the glass slides with hydrazine groups followed by the coupling with an aldehyde-peptide. Although this immobilization worked well, the preparation of the aldehyde-peptide by periodate oxidation of a N-terminal serine adds additional steps and we found it to be unsuitable when working with oxidation-sensitive peptides.

Recently, the Huisgen 1,3-dipolar cycloaddition reaction of azides with alkynes, known as the “click” reaction, has gained a lot of attention for modification and functionalization of biomolecules in and outside living systems [14,15]. Typically, these cycloaddition reactions are simple and easy to perform and are compatible with many functional groups. This has been demonstrated by the covalent and orthogonal attachment of biomolecules to solid surfaces to fairly rapidly prepare microarrays [16,17,18].

**Scheme 1:**
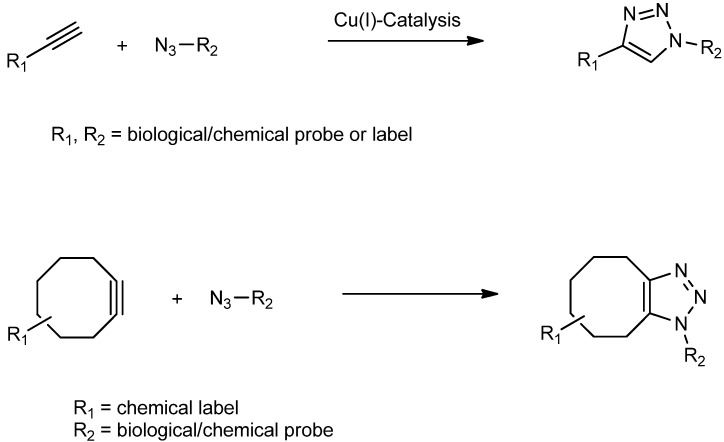
Huisgen 1,3-dipolar cycloaddition—copper(I) catalyzed reaction of terminal alkynes with azides (CuAAC) and copper-free strain-promoted alkyne-azide cycloaddition (SPAAC).

Originally, the Hüsigen 1,3-dipolar cycloaddition worked with heat, and later a copper(I) catalytic method was developed, which works well for many applications (Scheme 1). However, it was quickly realized that the cytotoxic effects of copper are an enormous drawback for many *in vivo* applications. To overcome this problem, the concept of strain-promoted alkyne-azide cycloaddition (SPAAC, Scheme 1), which does not require the use of any additional reagents was developed [19].

**Figure 1:**
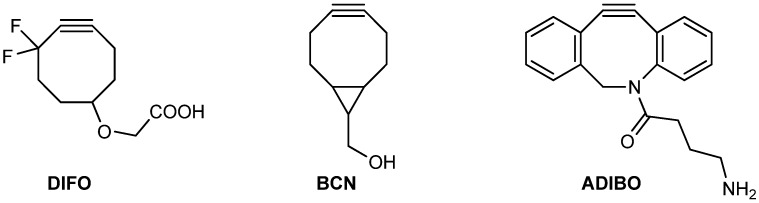
Difluorinated cyclooctyne (DIFO)-, bicyclo[6.1.0]nonyne (BCN)- and azadibenzocyclooctyne (ADIBO)-type SPAAC reagents.

Today, many cyclooctyne-based probes are available. Their reactivities and properties can be tuned by electronic and steric effects [19,20]. Introduction of fluorine atoms, benzo fusion or amide nitrogens to the cyclooctyne system increases the kinetics of the Huisgen 1,3-dipolar cycloaddition dramatically (see some examples in Figure 1). The reactivity increases from DIFO to BCN to ADIBO. Another important factor is the lipophilic properties of the cyclooctyne-probes, which may limit their water solubility and may lead to undesired hydrophobic interactions with peptides/proteins. From a chemist’s point of view, the ease of synthesis and overall yield also has to be considered in the choice of cyclooctyne-based probes.

The azadibenzocyclooctyne (ADIBO)-based click chemistry probes are a good compromise between reactivity and lipophilic properties. They can be easily prepared and are also commercially available. We previously exploited ADIBO-based strain-promoted alkyne-azide cycloaddition (SPAAC) described by Kuzmin *et al.* [16] to construct peptide microarrays for tumor auto-antibody (TAA) molecular diagnostic tests [21]. We also tested the copper-catalyzed click reaction (CuAAC) of azido-functionalized peptides with terminal acetylene-functionalized glass slides, but this immobilization approach was rather sluggish and did not give reproducible results (unpublished data) [22]. In the present study, we further describe characteristics of this catalyst-free click chemistry for microarray construction and the application of azide-modified blocking reagents to reduce non-specific binding, which usually is inevitable and detrimental to the analytical performance when working with serum samples. We also show that ADIBO-based click chemistry is compatible with automated nano-liter scale microarray fabrication.

## 2. Results and Discussion

### 2.1. Kinetics of Click Immobilization of Small Molecules and Peptides

**Scheme 2:**
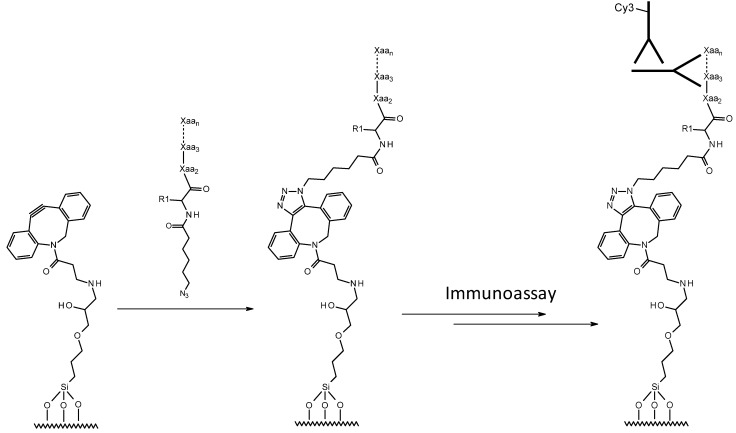
Schematic representation of covalent immobilization of peptides to azadibenzocyclooctyne (ADIBO)-activated glass slides via a copper-free azide-alkyne cycloaddition “click” chemistry reaction. Antigenic peptide baits capture anti-peptide antibodies which are detected with a fluorescently-labeled secondary antibody. Xaa denotes an unspecified amino acid and R_1_ is the side chain of the amino-terminal amino acid, which is a Cy5-labeled lysine in some peptides (see Table 1). Reprinted (adapted) with permission from Cosandey *et al*. [21] Copyright (2012) Swiss Chemical Society (Chimia).

As we had not studied copper-free click immobilization kinetics of azide-derivatized peptides to ADIBO-activated slides in detail before [21], we set out to investigate this further and prepare several azido-functionalized peptides with and without Cy5-label (Scheme 2, Table 1).

**Table 1:** Description of peptides used in this study and their chemical modifications.

Name	Sequence (amino- to carboxy-terminus)	Comment
N_3_-Cy5-myc	N_3_- 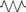 -K(Cy5)- EQKLISEEDL-OH	Labeled assay development model
N_3_-myc	N_3_- 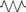 -EQKLISEEDL-OH	Assay development model
N_3_-P1	N_3_- 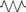 -DTQLKSRDPSKIPV-NH_2_	NC biomarker peptide
Cy5-P1	K(Cy5)- DTQLKSRDPSKIPV-NH_2_	Labeled NC biomarker peptide for characterization of NSB
N_3_-Cy5-P1	N_3_- 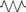 -K(Cy5)-DTQLKSRDPSKIPV-NH_2_	Labeled NC biomarker peptide
N_3_-P2	N_3_- 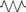 -*Undisclosed sequence*	Putative cancer biomarker peptide
N_3_-Cy5-P2	N_3_- 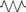 -K(Cy5)-*Undisclosed sequence*	Labeled putative cancer biomarker peptide

N_3_-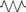
- corresponds to N_3_-(CH_2_)_5_-(CO)-, NC = negative control.

**Figure 2:**
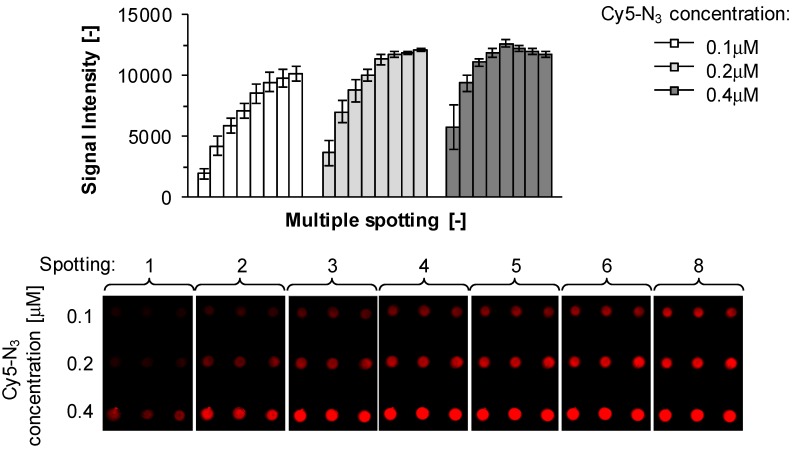
Characterization of slide reactivity. Fluorescence signal intensity at λ = 635 nm recorded on ADIBO-activated glass slides after sequential spotting (8 times) of 2 μL of Cy5-N_3_ in 25% DMSO/H_2_O at three different concentrations (0.1, 0.2 and 0.4 μM, respectively) and corresponding wash cycles (*top*). A microarray image is shown from one representative experiment (*bottom*). Data are mean values ± SD of triplicate measurements.

First, Cy5-N_3_ at 0.1 mM concentration in H_2_O was tested and saturation fluorescence intensities were reached within 5 min (unpublished results) [23] which appeared significantly faster than the 100 min Kuzmin and coworkers [16] had observed with Oregon green azide. Repetitive cycles of slide spotting and washing showed that a higher plateau-level fluorescence intensity value was only slightly dependent on spotting concentrations (Figure 2).

**Figure 3:**
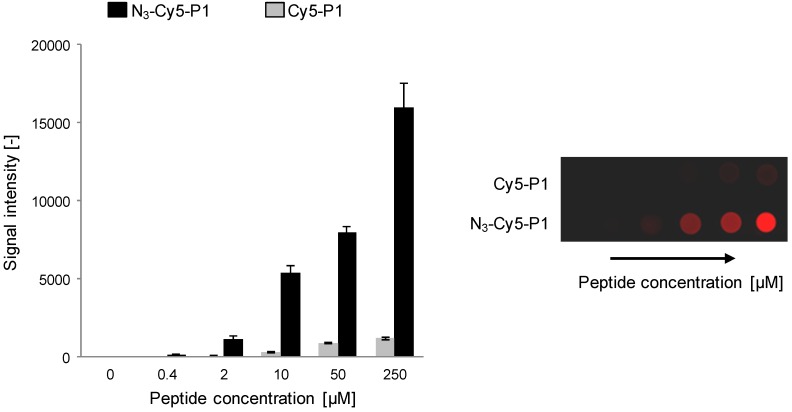
Immobilization reaction specificity of peptide N_3_-Cy5-P1 versus non-specific binding (NSB) of peptide Cy5-P1 to ADIBO-functionalized glass slides. 1 μL of peptides of various concentrations were spotted. Fluorescence signal was recorded at λ = 635 nm after successive slide washing (*left*). A corresponding microarray image from one representative experiment is shown (*right*). Experiments were performed three times and data are mean values ± SD of triplicate measurements.

To verify this increase in signal intensity, particularly when working with peptides, was not due to residual, non-specifically bound reagent (*i.e*., insufficiently thorough washes), we compared the fluorescence signal evolution for peptide N_3_-Cy5-P1 *versus* peptide Cy5-P1 lacking the azido functionality. A marginal fluorescence signal increase was observed for Cy5-P1 at high spotting concentrations (>50 mM, see Figure 3) which are not practical (and necessary) for microarray production, indicating a negligible degree of non-specific, physical adsorption.

**Figure 4:**
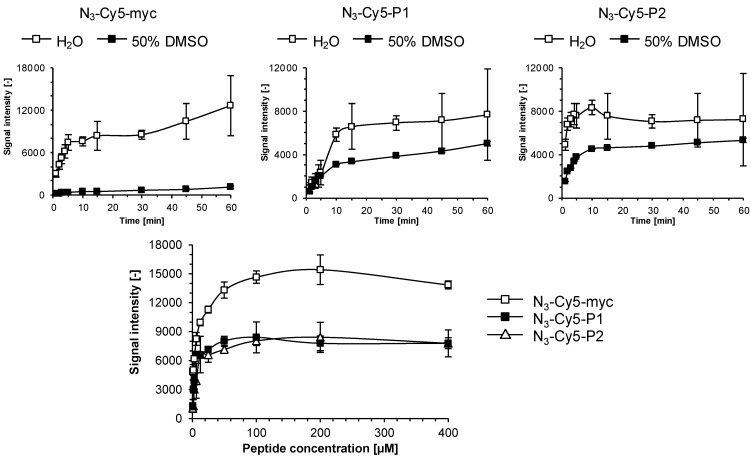
Reaction kinetics of azido-functionalized peptides to ADIBO-modified glass slides. 1 μL of peptides N_3_-Cy5-myc, N_3_-Cy5-P1 and N_3_-Cy5-P2 diluted in H_2_O or H_2_O:DMSO at a 1:1 ratio were deposited and allowed react for the indicated time. Fluorescence signals were recorded at λ = 635 nm after slides washing (*top*). Determination of peptide saturation concentrations (*bottom*): 1 μL of peptides N_3_-Cy5-myc, N_3_-Cy5-P1 and N_3_-Cy5-P2 diluted in H_2_O at indicated concentrations were spotted and incubated for 30 min to allow immobilization, washed extensively for 3x three min with PBS 1X containing 0.05% of Tween-20, rinsed with H_2_O and dried under N_2_-flux. Kinetic and saturation experiments were performed twice and data are mean values ± SD of triplicate measurements.

Peptides N_3_-Cy5-myc, N_3_-Cy5-P1 and N_3_-Cy5-P2 attained 80% of maximum signal within 10 min at room temperature which shows that larger and more complex molecules than Oregon green azide and Cy5-N_3_ react more slowly, but still fast enough from a microarray fabrication point of view (Figure 4, *top*). It is noteworthy that the solvent has a significant impact on kinetics and attainable fluorescence signal plateaus. Peptide N_3_-Cy5-P1, which has the same amino acid composition as N_3_-Cy5-P2, but in a scrambled sequence, reaches intensities of approximately 8,000 in water compared to 5,000 in 50% DMSO/H_2_O. In contrast, the more polar undecapeptide N_3_-Cy5-myc containing several negatively charged residues reacted very poorly in 50% DMSO/H_2_O presumably due to solubility constraints.

To investigate optimal substrate concentrations for the ADIBO-based SPAAC reaction we spotted ADIBO-modified slides and incubated for 10 min at room temperature with varying concentrations of the peptides N_3_-Cy5-myc, N_3_-Cy5-P1 and N_3_-Cy5-P2 in H_2_O or in H_2_O:DMSO at a 1:1 ratio, ranging from 0.1 to 400 μM. The integral fluorescence signal intensities recorded (Figure 4, *bottom*) showed that signals of 7,000 and 10,000 were generated from solutions of just 20 μM and saturation of Cy5-fluorescence occurred at 100 μM.

### 2.2. Immunoassay Performance Comparison

**Figure 5:**
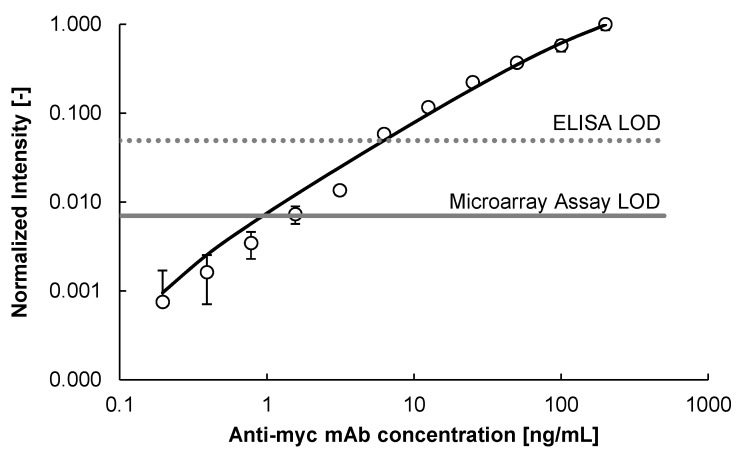
Anti-*myc* monoclonal antibody titration experiments: N_3_-*myc* peptide diluted to 200 μM in 50% DMSO/H_2_O was immobilized onto ADIBO-functionalized glass slides for 30 min. Unreacted ADIBO groups were capped with 6-azido-hexanoic acid (**iHxa**, see Table 2) at a concentration of 1.3 mM for 30 min. Immunodetection was performed with different anti-*myc* antibody concentrations and with Cy3-labeled detection antibody at a concentration of 2 μg/mL. Fluorescence signals were recorded at λ = 532 nm. Data are mean values of triplicate measurements ± SD. The LOD for the peptide microarray assay and MTP-based ELISA are indicated with a straight and a dotted grey line, respectively. LOD was calculated as follows: mean background signal plus three times standard deviation of the background signal. Curve fitting is based on the 4-parameter non-linear logistic regression function [24].

To assess assay limit-of-detection (LOD) of the anti-*myc*-peptide monoclonal antibody (mAb) we conducted a comparative study similar to earlier experiments using the BRCA-1 associated RING domain 1 (BARD1) cancer biomarker system [21]. The goal was to benchmark the peptide microarray platform performance against the classic ELISA format one. The *myc*-peptide microarray fabricated both on the microliter as well as the nanoliter scale with the ADIBO-based click chemistry showed a superior LOD of 2 ng/mL compared to a streptavidin microtiter plate (MTP) and biotinylated *myc*-peptide-based ELISA, for which a LOD of 7 ng/mL was obtained (Figure 5). These findings confirm earlier results where the LOD for the mouse anti-BARD1 antibody was lower for the peptide microarray platform compared to the ELISA format on a microliter [21] and nanoliter scale (unpublished results) [23].

**Figure 6:**
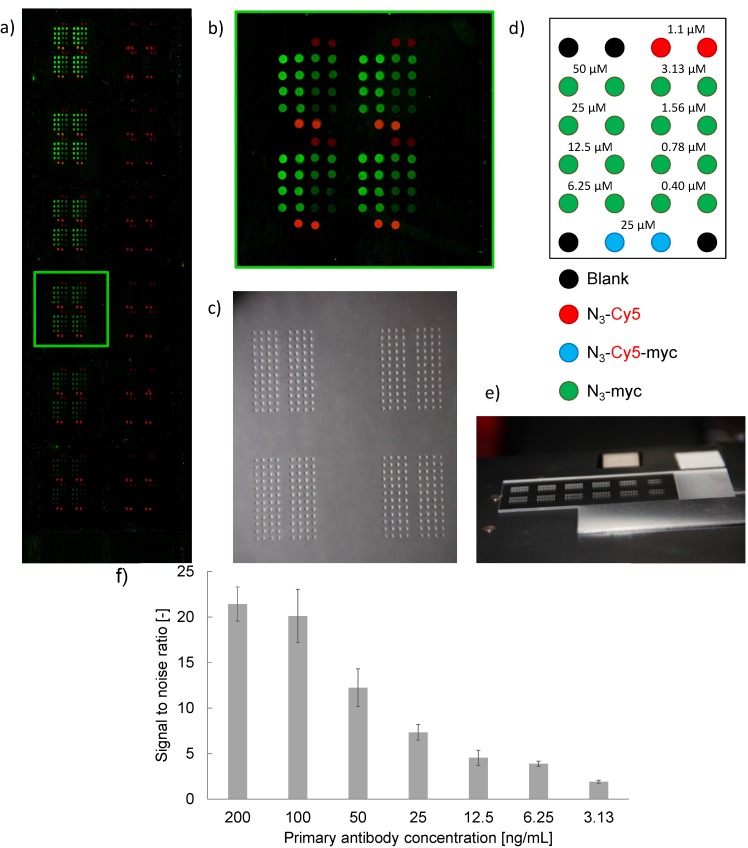
Image of anti-*myc* antibody titration experiment with 1 nL of N_3_-*myc* peptide deposited at concentrations according to the layout in (**d**) and probed with anti-*myc*-peptide mAb in a 1152-spot array at concentrations of 200, 100, 50, 25, 12.5, 6.25, 3.13, 1.56, 0.78, 0.39, 0.2 and 0.1 ng/mL (**a**, top to bottom, left to right). (**b**) sub-array image at an anti-*myc* mAb concentration of 25 ng/mL. (**c**) microscopy image of slide section with droplets, (**e**) photo of spotted glass slide with 12 sub-arrays visible. (**f**) Signal-to-noise (background) ratios (SNR) for spots at a deposited peptide concentration of 25µM and anti-*myc* mAb concentrations above the LOD, *i.e.* between 200 and 3.13 ng/mL. Spot diameter (mean value, SD) = 273 ± 20 μm (n = 64 spots); spot homogeneities, CV = 18% (n = 64 spots); noise (background) intensity (mean value, SD) = 441 ± 116 [a.u.].

Figure 6 illustrates the robustness of the ADIBO-based click chemistry in combination with an automated piezoelectric nanoliter scale spotting device. Positional accuracy, spot size variability and intra-spot fluorescence intensity homogeneity and inter-spot fluorescence intensity reproducibility are exceptionally good. ADIBO glass slide activation and functionalization with various fluorescent reagents including modified peptides were shown to produce homogenous surfaces and reproducible fluorescent intensities (data not shown), essential from a microarray manufacturing perspective. It should also be noted that the actual printing process of the slides takes as little as three minutes and the total volume of peptide solution needed is approximately 48 nL per slide for each of the 24 channels. These performance characteristics suggest the potential for high productivity in peptide microarray preparation.

### 2.3. Blocking Reagents to Reduce Non-Specific Binding (NSB)

Multivariate serological assays that detect for instance multiple tumor auto-antibodies (TAA) against aberrant tumor-associated antigens often suffer limited sensitivity and specificity. It was shown recently that detection levels depend strongly on microarray surface chemistry [25]. Patient sera are heterogeneous, complex sample matrices that tend to give high background signals due to NSB of serum components to the solid surface. Frequently this is partially addressed via addition of bovine serum albumin (BSA) or fetal calf serum (FCS) to reduce uncontrolled physical adsorption via a competition mechanism.

**Figure 7:**
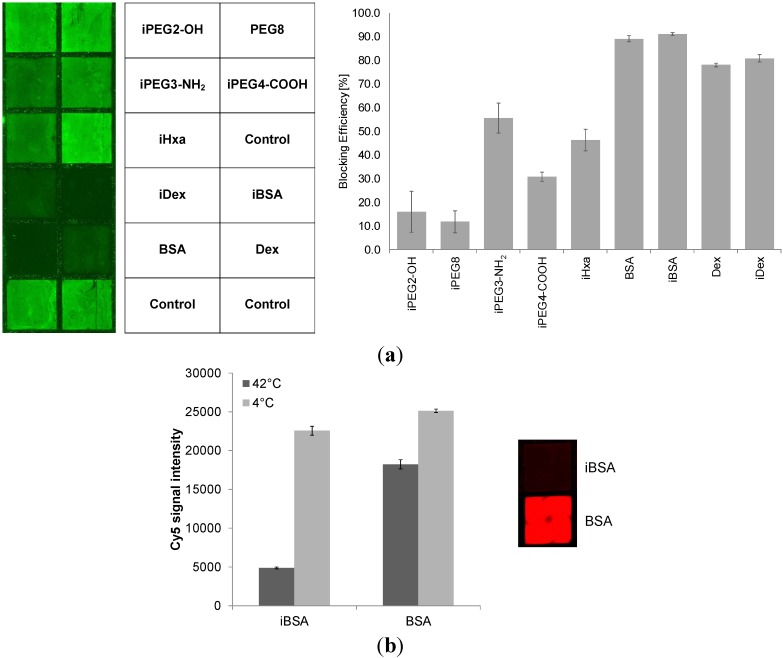
(**a**) Comparison of blocking efficiency of immobilized azide derivatives at concentrations of 100 µM for the different PEG-azide reagents and 10 µM for dextran, azide-modified dextran, BSA and azide-modified BSA tested against Cy3-labeled goat anti-rabbit IgG. The non-specific binding (NSB) is made visible as *green* squares. Blocking efficiency is given as percentage [%] of signal reduction of control wells that have ADIBO-functionalized slides surfaces. Data are mean values ± SD of hexaplicate experiments. Fluorescence signal intensities across control wells were 2,680 ± 165 and across iBSA wells 230 ± 22. (**b**) Comparison between physically adsorbed and covalently attached BSA. The use of azide-modified BSA allows nearly complete capping of the free ADIBO groups. Physically adsorbed BSA cannot prevent Cy3-N_3_ to penetrate the layer and react with the ADIBO groups as visualized with the red squared well. Higher temperatures increase the coupling rates of BSA-N_3_ and also appear to slightly improve surface adsorption of unmodified BSA to decrease Cy5-N_3_ permeability. Data are mean values ± SD of quadruplicate experiments.

Another approach involves the capping of unoccupied binding sites with blocking reagents to minimize NSB. For example, various poly(ethylene glycols) (PEG) have been reported to reduce NSB of proteins [26,27,28,29]. Surfaces modified with diamino-PEG 2000 (DAPEG) have been shown in another study to resist non-specific adsorption of horseradish peroxidase-conjugated streptavidin (SA-HRP) [30]. Kuzmin *et al.* [16] reported that PEGylation of the glass slides, e.g. blocking with ADIBO-PEG_4_-amine via click chemistry, had a pronounced effect in reducing or completely eliminating non-specific binding. In this work the PEG-modified surface prevented avidin from binding. To investigate this further we modified ADIBO-activated slides surfaces with 6-azidohexanoic acid, several commercially available PEG-azides and both azide-modified BSA as well as dextran prepared in our laboratory (Figure 7 and Table 2).

**Table 2:** Overview of surface immobilized blocking reagents used to study efficacy in reducing IgG induced NSB to modified microarray surfaces (prefix “i” denotes an “immobilized” molecule).

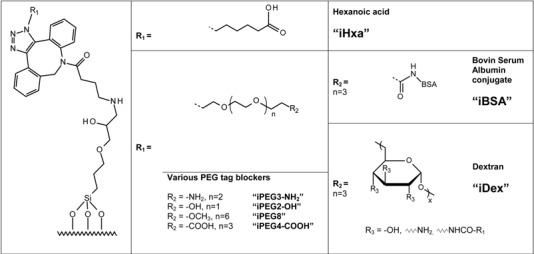

Dextrans [31], aminodextrans [32] and carboxydextrans [33] can protect surfaces from protein unspecific adsorption. We indeed observed that iBSA and iDex blocked NSB of Cy3-labeled IgG much more strongly than iHxa and the PEG-derivatives. iBSA and iDex blocking efficiencies were over 90% and 80%, respectively, whilst the iPEG blocking reagent variants, with the exception of iPEG4-NH_2_, were 30% effective or less. Interestingly, non-modified BSA and dextran showed comparable NSB prevention capacities to their azide-modified counterparts, presumably due to their physical adsorption to the apolar ADIBO groups. However, as can be seen in Figure 7(b), non-modified BSA, in contrast to iBSA was not capable of preventing Cy5-N_3_ from reacting with the ADIBO-groups, indicating permeability of the adsorbed BSA layer for small molecules. When working with complex serum samples containing multiple smaller to larger molecular components, covalently attached BSA and dextran can prove advantageous: smaller, auto-fluorescent molecules are less likely to penetrate and be able to adsorb to the microarray surface giving rise to elevated non-specific fluorescence signals. Covalently anchored blocking reagents such as BSA and dextran are expected also to tolerate more stringent assay-related wash cycles possibly resulting in better signal to noise ratios (SNR) crucial for good analytical sensitivities.

## 3. Experimental

### 3.1. General information, Peptide Synthesis and Modifications

6-Azidohexanoic acid was prepared according to literature [34]. All chemicals and solvents were reagent grade and used as supplied. The glass slides were functionalized as previously published [21] and stored at −20 °C. A negative control 14 amino acid peptide (P1) was synthesized in-house using Fmoc-protected amino acids and a standard coupling procedure on an Apex DCFWS 396 synthesizer (AAPPTec, Louisville, KY, USA). Custom peptide P2 and the *myc*-peptide were ordered crude on resin from Genscript (Genscript, NJ, USA). Derivatization with 6-azidohexanoic acid and Cy5-labeled lysine (P/N: 5055, AAT Bioquest, CA, USA) was performed using the same chemistry. Purification was performed on all products by preparative RP-HPLC, following which products were lyophilized and finally their molecular weights and sequences were verified by mass spectroscopy (Agilent UPLC-qTOF 6530, Santa-Clara, CA, USA). N_3_-P1 peptide expected mass is 1,722.9 g/mol, measured ions were 1,723.9 *m/z* (z = 1) and 862.6 *m/z* (z = 2). Cy5-P1 peptide expected mass is 2,349.1 g/mol, measured ions were 1,175.5 *m/z* (z = 2) and 784.0 *m/z* (z = 3). N_3_-Lys(Cy5)-P1 peptide expected mass is 2,489.2 g/mol, measured ions were 1,245.7 *m/z* (z = 2) and 830.7 *m/z* (z = 3). N_3_-P2 peptide expected mass is 1,722.9 g/mol, measured ions were 1,723.9 *m/z* (z = 1), 862.0 *m/z* (z = 2) and 575.0 *m/z* (z = 3). N_3_-Lys(Cy5)-P2 peptide expected mass is 2,489.2 g/mol, measured ions were 1,245.6 *m/z* (z = 2) and 830.8 *m/z* (z = 3). Purity was assessed by UPLC-UV as >95%. N_3_-*myc* peptide expected mass is 1,341.4 g/mol, measured ions were 1,342.7 *m/z* (z = 1) and 671.9 *m/z* (z = 2). N_3_-Lys(Cy5)-*myc* peptide expected mass is 2108.8 g/mol, measured ions were 1,055.5 *m/z* (z = 2) and 704.0 *m/z* (z = 3). Purity was assessed by UPLC-UV as >80%.

### 3.2. Blocking Reagents

Commercially available reagents were directly diluted in DMSO/H_2_O = 1:1 to achieve a concentration of 351 µM for the amino-PEG3-azide (P/N: CLK-L011-10, Jena Bioscience, Jena, Germany), 3.5 mM for the carboxylic-PEG4-azide (P/N: CLK-AZ-102, Jena Bioscience), 3.5 mM for the hydroxyl-PEG2-azide (P/N: CLK-L004, Jena Bioscience), 3.5 mM for the PEG8-azide (P/N: CLK-L003, Jena Bioscience) and 1.3 mM for 6-azidohexanoic acid.

BSA (P/N: 10 735 078 001, Roche Diagnostics, Rotkreuz, Switzerland) and aminodextran (P/N: D1861, Life Technologies, Grand Island, NY, USA) derivatization was prepared according to Piercenet amine reactive azide-labeling reagent NHS-PEG4-N_3_ (P/N: 26130, Piercenet, Rockford, IL, USA) and purified with 3 mL “Slide-A-Lyzer” dialysis cassettes 10K MWCO (P/N: 66810, Piercenet). Stock solutions of N_3_-BSA were made at 7.1 mg/mL and N_3_-dextran at 9.1 mg/mL in PBS. Coupling was controlled with UPLC/MS analysis on UPLC 1290 and qTOF 6530 from Agilent. Unmodified BSA was observed after deconvolution (maximum entropy) at 66,430 Da and coupled BSA at 70,300 Da resulting in a ratio of 12 to 13 azides per BSA. Synthesis of N_3_-dextran was evaluated by its ability to cap free ADIBO groups on activated slides in comparison of non-modified dextran.

### 3.3. Sequential Microarray Spotting

On a functionalized slide, 2 µL of Cy5-N_3_ (P/N: CLK-CCA-9295-1, Jena Bioscience) in 25% DMSO/H_2_O at concentrations of 0.1, 0.2 and 0.4 µM were manually dispensed with a 0.5–10 µL pipette to form spots of 2 to 3 mm diameter. Spots were then allowed to dry at room temperature for at least 10 min before stringent washing of the slides with methanol, followed by acetone and then water to insure proper elimination of non-covalently attached Cy5 prior to scanning. Once the recording operation had finished the procedure was repeated eight times.

### 3.4. Blocking Efficiency

A functionalized slide was installed in a Nexterion IC-16 hybridization chamber and 100 µL of blockers at 100 µM for PEG-derivatives or 10 μM for BSA and dextran were added. The chamber was maintained at 42 °C for 2h to let the molecules react with the ADIBO. Wells were washed four times with 300 µL of PBS + 0.05% Tween 20 prior to adding 100 µL of Cy3-labeled goat anti-rabbit IgG (P/N: 072-01-15-06, KPL, Gaithersburg, MD, USA) at 20 μg/mL in PBS and incubation for 30 min at room temperature. Washes were then performed and the hybridization chamber was opened, the slide was washed in a bath of PBS + 0.05% Tween 20 followed by a bath of deionized H_2_O and dried with a flow of N_2_ prior fluorescence scanning.

### 3.5. Fluorescence Immunoassay

One nanoliter of the corresponding peptide solution at concentrations of 50 to 0.4 µM was deposited with an automated microarray pattern printing system (see Section 3.7.) on activated ADIBO-slides to form spots of approximately 270 μm diameter. Once the spots had fully dried, slides were immersed in a bath of 50 mL of 6-azidohexanoic acid at a concentration of 1.3 mM for 30 min. Slides were subsequently washed in a falcon tube with phosphate buffer + 0.05% Tween 20 (PBS-T) and then H_2_O to remove peptides that had not reacted covalently. Slides were dried under a N_2_ flow and assembled into Nexterion IC-16 hybridization chambers. Wells were first blocked with a 5% BSA solution for 1 h. Between assay steps wells were washed three times with PBS-T + 0.05% Tween 20 to remove reagent excess. Primary antibody solution was prepared in PBS with 1% BSA at concentrations ranging from 0.4 to 200 ng/mL for mouse anti-*myc* peptide (P/N: M4439, Sigma-Aldrich, St. Louis, MO, USA). One hundred µL were dispensed into the wells and incubated for 30 min. at room tempearture. The last step involved 30 min. reaction with 100 µL of Cy3-labeled goat anti-mouse antibody (P/N: 072-01-18-06, KPL) at a concentration of 2 µg/mL. Slides were finally washed with PBS 1X + 0.05% Tween 20, hybridization chambers were dismantled and the slides briefly rinsed with ultrapure water and then dried with a flow of N_2_ or air before fluorescence measurements.

### 3.6. Streptavidin-Biotin ELISA

An indirect ELISA was developed in streptavidin pre-coated 96 well plates (P/N: 436014, Nunc Thermo Fisher, Waltham, MA, USA). In each well, 100 µL of a solution of 12.5 µg/mL biotin-*myc* (Biotin-Ahx-EQKLISEEDL, Genscript, NJ, USA) in PBS + 1% BSA was added. After a reaction time of 1 h at room temperature, the plate was washed with PBS + 0.05% Tween 20three times. Then 100 µL of 0.4 to 200 ng/mL mouse anti-*myc* peptide antibody (P/N: M4439, Sigma-Aldrich) in a PBS + 1% BSA solution was added in triplicate. After 1 h, the plate was washed again and 100 µL of goat anti-mouse IgG (H+L) phosphatase-labeled antibody (P/N: 074-1806, KPL) at 1 µg/mL was dispensed in each well. One hour later, the plate was washed a final time and 150 µL of 0.2 mg/mL 4-nitrophenyl phosphate (P/N: 71768, Sigma-Aldrich) substrate in DIEA/MgCl_2_ buffer at pH 9.6 were added. After 20 min. absorbances were measured on a Molecular Devices (Sunnyvale, CA, USA) Paradigm microtiter plate reader at 405 nm.

### 3.7. Automated Microarray Pattern Printing

Functionalized nano-spotted slides were printed according to a published method [16] by ejecting approximately 1 nL microdrops by a system developed at HEPIA and validated in a recent study [35].

### 3.8. Microarray Spot Fluorescence Measurements

Slides were measured with a non-confocal GenePix 4000B microarray scanner (Molecular Devices) in simultaneous 2-channel scanning mode (excitation at λ = 532 nm, emission range from 558 to 593 nm and excitation at λ = 635 nm, emission range from 660 to 690 nm). Pixel resolution was set at 10 μm unless otherwise stated. Dual photomultipliers (PMTs) with manual gain adjusted for better dynamic range were used for data acquisition and data were stored as TIFF 16-bit files. Features were manually extracted and the averages for each spots were used for the analysis.

## 4. Conclusions

A series of azide-derivatized and some cyanine fluorescent dye-labeled, peptides and azide-modified dextran and BSA have been prepared. Excellent immobilization kinetics to azadibenzocyclooctyne (ADIBO)-activated glass slides for the manufacturing of miniaturized high-density peptide microarrays has been shown. The same catalyst-free click chemistry was employed to covalently attach various polyethylene glycol (PEG) type derivatives. However, the experiments suggest that both immobilized BSA and dextran are superior in terms of reducing non-specific binding (NSB) when working with Cy3-labelled IgG. Further studies with other proteins such as human serum albumin and immunoglobulins IgA and IgM and ultimately with whole serum from healthy individuals and patients are necessary to validate the efficiencies of immobilized blocking reagents to shield against unspecific adsorption. Serological assay sensitivity requirements demand excellent signal-to-noise ratios (SNR), hence low background signal levels. As shown, strain promoted alkyne-azide cycloaddition (SPAAC) reactions of both azide-derivatized peptides and blocking reagents with cyclooctyne-activated glass slides present a robust and efficient fabrication process of microarray-based tumor auto-antibody (TAA) molecular diagnostic tests which may prove suitable for use with serum samples.
